# A new highly specialized cave harvestman from Brazil and the first blind species of the genus: *Iandumoema smeagol* sp. n. (Arachnida, Opiliones, Gonyleptidae)

**DOI:** 10.3897/zookeys.537.6073

**Published:** 2015-11-18

**Authors:** Ricardo Pinto-da-Rocha, Rafael da Fonseca-Ferreira, Maria Elina Bichuette

**Affiliations:** 1 Departamento de Zoologia, Instituto de Biociências da Universidade de São Paulo, Rua do Matão, travessa 14, 321, 05508-090, São Paulo, SP, Brazil; 2 Departamento de Ecologia e Biologia Evolutiva, Universidade Federal de São Carlos, Rodovia Washington Luis, km 235, PO Box 676, 13565-905, São Carlos, SP, Brazil; 3 Programa de Pós-graduação em Biologia Comparada, Universidade de São Paulo, Avenida dos Bandeirantes 3900, 14040-901, Ribeirão Preto, SP, Brazil

**Keywords:** Endemism, troglobitic, limestone, Espinhaço Supergroup, Minas Gerais state

## Abstract

A new species of troglobitic harvestman, *Iandumoema smeagol* **sp. n.**, is described from Toca do Geraldo, Monjolos municipality, Minas Gerais state, Brazil. *Iandumoema smeagol* **sp. n.** is distinguished from the other two species of the genus by four exclusive characteristics – dorsal scutum areas with conspicuous tubercles, enlarged retrolateral spiniform tubercle on the distal third of femur IV, eyes absent and the penial ventral process slender and of approximately the same length of the stylus. The species is the most highly modified in the genus and its distribution is restricted only to caves in that particular area of Minas Gerais state. The type locality is not inside a legally protected area, and there are anthropogenic impacts in its surroundings. Therefore, *Iandumoema smeagol* **sp. n.** is vulnerable and it must be considered in future conservation projects.

## Introduction

The subterranean or hypogean fauna is ecologically categorized according to the degree of the populations’ dependence and specialization to that environment, as proposed by Schiner (1854) and modified by Racovitza (1907) (*apud* Barr and Holsinger 1985, Trajano 2012): trogloxenes, organisms which are regularly found in caves, but that periodically return to the surface to feed and often to reproduce; troglophiles, organisms that can complete their life-cycle in either environment; and troglobites, organisms restricted exclusively to caves. Troglobites have evolved isolated in a peculiar selective regime, distinct from their ancestrals’: total absence of light, a tendency to environmental stability, lack of primary production and low energy intake (Culver and Pipan 2009). In order to survive and effectively colonize the hypogean realm, subterranean species must reproduce, defend their territories and find food and mates in this environment, regardless of vision (Gibert and Deharveng 2001). Several specializations related to the life in subterranean environment have been reported in literature – the autapomorphies, called troglomorphisms (Christiansen 2012).

In caves, harvestmen are found near to or in association with organic matter deposits or spots, under blocks and rocks, on the walls, and on the ceiling, exhibiting solitary or gregarious behavior (Reddell 2012). To date, eight species of troglobitic harvestmen have been described in Brazil, belonging to the families Gonyleptidae Sundevall, 1833 (seven species; one Pachylospeleinae and six from Pachylinae subfamily) and Escadabiidae Kury and Pérez 2003 (one species), in addition to several troglophile and trogloxene representatives (Trajano and Bichuette 2009, Willemart and Taques 2013). At least six other undescribed species has been reported as restricted to subterranean environments (Hara and Pinto-da-Rocha 2008, Willemart and Taques 2013).

The gonyleptid genus *Iandumoema* Pinto-da-Rocha 1996, comprises two strictly subterranean species (troglobitic) up to now: *Iandumoema uai* Pinto-da-Rocha 1996 and *Iandumoema setimapocu* Hara and Pinto-da-Rocha 2008. The genus belongs to the polyphyletic Pachylinae (Pinto-da-Rocha et al. 2014) and its distribution is restricted to northern Minas Gerais state (eastern Brazil): *Iandumoema setimapocu* is endemic to only one cave (Lapa do Zu cave, municipality of Coração de Jesus) (Hara and Pinto-da-Rocha 2008) and *Iandumoema uai* is restricted to two caves (Gruta Olhos d’Água and Lapa do Cipó caves, municipality of Itacarambi) (Pinto-da-Rocha 1996, Monte et al. in press).

A new cave species of *Iandumoema* is herein described, being the second troglobitic harvestman with no eyes for Brazil (the first being the Gonyleptidae *Giupponia chagasi* Perez and Kury 2002, from Serra do Ramalho karst area, Bahia state, northeastern Brazil). This record corroborates the hypothesis of an exclusively troglobitic genus.

## Material and methods

### Study area

**Figure 1.**
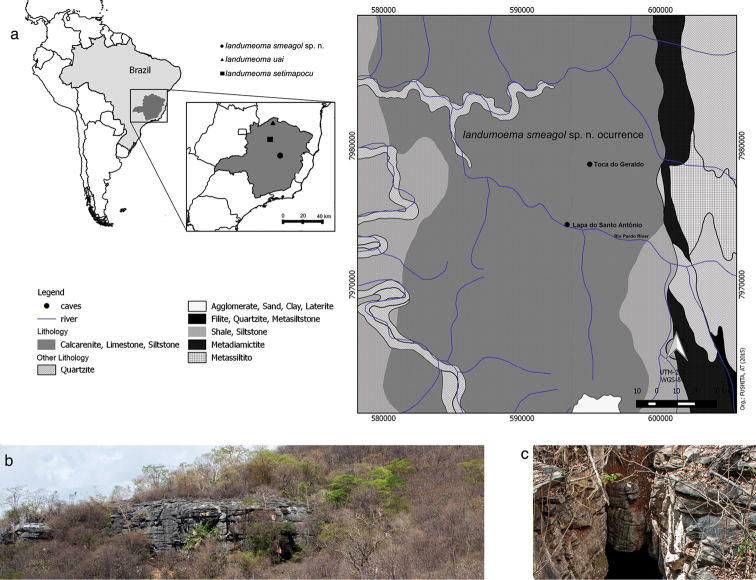
**a** map of the study area at Monjolos municipality, Minas Gerais state, Brazil **b** Karst relief of Monjolos regions **c** entrance of Toca do Geraldo cave, a limestone cave of Bambuí Geomorphological Unit.

*Iandumoema smeagol* sp. n. is recorded from two caves from Monjolos region, Minas Gerais State, Brazil. This region is located in the central east part of the southern portion of the São Francisco Craton, Velhas river basin, with a mean altitude of approximately 600 meters, inserted in the Sete Lagoas Formation, Bambuí Group, which has a relief typical of karst carbonate regions (Stávale 2012, Guimarães 2012) (Figure 1a). Monjolos region is characterized by evident karst relief, marked by large limestone cliffs, karrens, dolines, sinks, and resurgences, representing the exokarst (Figure 1b), and subterranean watercourses, diverse speleothems and caves, representing the endokarst (Guimarães 2012). According to the Köppen-Geiger climatic classification, the region has a tropical climate with a dry season (Kottek et al. 2006) type Aw (Sá Junior et al. 2012), with mean annual temperatures ranging between 20 and 21 °C. The vegetation is dominated by plants of the ‘cerrado’ *sensu strictu*, cerrado fields, and seasonal forests (Guimarães 2012). However, the vegetation surrounding the cave is under anthropogenic actions, such as pasture and agricultural activities.

Toca do Geraldo is a limestone cave which extends approximately 1.5 km, with one entrance in a crack (Figure 1c) and another in the ceiling and a subterranean stream, which extends at least 400 meters. The harvestmen were found on the wet walls and sometimes in the silt substrate, next to the drainage, always in the aphotic zone. This cave has guano piles and litter as main food source for other cave arthropods such as crickets, cockroaches, mites, etc. Because the perennial drainage, the humidity is high (higher than 70%), even during the dry season. Lapa do Santo Antonio is also a limestone cave *ca.* 4.6 km far from Toca do Geraldo and also possess a subterranean stream; however, is an impacted cave due uncontrolled visitation. This cave has *ca.* of 300 m of extension.

## Methods

The type material were collected, fixed in 70% ethanol and examined under a stereomicroscope. Live specimens were collected to observe the coloration *in vivo*. We took photographs and length measurements using a Leica stereomicroscope (M205C). Methods and terminology follow Acosta et al. (2007). The pattern of the macrosetae of the penis follows Kury and Villarreal (2015). Coloration is based on specimens immersed in ethanol and living specimens. Abbreviations used in Table 2 are: Tr = trochanter; Fe = femur; Pt = patella; Ti = tibia; Mt = metatarsus; Ta = tarsus. All measurements are in millimeters. The types are deposited in the Museu de Zoologia, Universidade de São Paulo, São Paulo (MZUSP) and Laboratório de Estudos Subterrâneos, Universidade Federal de São Carlos, São Carlos (LES/UFSCAR).

In the natural habitat, through *ad libitum* method (Altmann 1974), the behavior and spatial distribution were observed. On four occasions, the minimal abundance through visual census method (Krebs 1999) was recorded, covering an extension of 300 m. Measurements of temperature and air humidity were recorded through a thermo-hygrometer.

## Results

### Key for the male of *Iandumoema*

**Table:** 

1	Apophysis of coxa IV directed obliquely backwards (parallel to body main axis)	**2**
–	Apophysis of coxa IV directed laterally (perpendicular to body main axis)	***Iandumoema uai***
2	Dorsal scutum areas with conspicuous tubercles (paramedian pair higher than wide), retrolateral trochanter IV with larger tubercle on apex	***Iandumoema smeagol* sp. n.**
–	Dorsal scutum areas with low tubercle (as heigh as wide), retrolateral trochanter IV without larger tubercle on apex	***Iandumoema setimapocu***

#### Iandumoema smeagol sp. n.

Taxon classificationAnimaliaOpilionesGonyleptidae

http://zoobank.org/AAFD82A9-FA5B-4A71-958A-1150951CB142

Figures 2 , 3 , 4 , 5 , 6 , 7 , 8 , 9 , 10 , 11 , 12 , 13

##### Type material.

Male holotype, Brazil, Minas Gerais, Monjolos, Toca do Geraldo cave, S18°16'43.31", W44°06'10.96’, 08.VII.2014, R. Fonseca-Ferreira, M.E. Bichuette, I. Arnone and J.E. Gallão leg. (MZUSP 67946). Paratypes: same locality of holotype, 22.II.2014, Rafael Fonseca-Ferreira and B.G.O. do Monte leg., one male (LES/UFSCar 0006298); Brazil, Minas Gerais, Lapa do Santo Antônio cave, S18°19'07,65", W44°07'03.32’, 21.II.2014, Rafael Fonseca-Ferreira and B.G.O. do Monte leg., one female (LES/UFSCar 0006299); same locality of holotype, 22.II.2014, Rafael Fonseca-Ferreira and B.G.O. do Monte leg., two male (MZUSP 67947 and MZUSP 67948).

##### Etymology.

The specific epithet refers to the hobbit named Smeagol, created by J.R.R. Tolkien, being the original name of Gollum – the dweller of the caves located below the Misty Mountains of Middle-earth of the Lord of the Rings book.

##### Diagnosis.

*Iandumoema smeagol* sp. n. can be distinguished from other *Iandumoema* species by the following exclusive characteristics: dorsal scutum areas with conspicuous tubercles (paramedian pair much higher than wide), enlarged retrolateral spiniform tubercle on the distal third of femur IV, eyes absent and the penial ventral process slender and of approximately the same length as the stylus – and by the combination of the following characters: four pairs of macrosetae on penial basal group A+B (six in *Iandumoema uai*), three or four pairs of macrosetae on penial distal group C (six pairs in *Iandumoema uai*); and the apex of the penial truncus narrower than ventral plate basal width (wider in *Iandumoema uai*), and the setae of male pedipalpal tibia ectally and mesally with IiIi (ectally with IiiIi and mesally with IiIi in *Iandumoema setimapocu*). A more detailed comparison of morphological and meristic features of *Iandumoema* species are provided in Table 1.

**Table 1.** Comparative morphological and meristic data for the three *Iandumoema* species from Brazilian caves (adapted from Hara and Pinto-da-Rocha 2008).

Characters	*Iandumoema uai* Pinto-da-Rocha, 1996	*Iandumoema setimapocu* Hara & Pinto-da-Rocha, 2008	*Iandumoema smeagol* sp. n.
Eyes condition	At least twice the diameter of tubercles on carapace	Same or similar size of diameter of tubercles on carapace	Absent
Setae on male pedipalpal tibia	Ectally and mesally with IiIi	Ectally with IiiIi and mesally with IiIi	Ectally and mesally with IiIi
Direction of dorso-apical apophysis on male coxa IV	Backwards and laterally	Obliquely backwards, close to body	Obliquely backwards, close to body
Submedian prolateral apophysis on male trochanter IV	Absent	Present	Present
Large tubercles on dorsal male femur apex	Two (one prolaterally, the other retrolaterally)	Three (two as in *Iandumoema uai*, plus a large median one)	Two (one prodorsal and one median)
Number of pair of macrosetae on penial basal group (A+B)	6	4	4
Number of pairs of macrosetae on penial distal group (C)	3	4	3–4
Shape of penial ventral process	Short and serrate	Short and serrate	Slender and approx. same length as stylus, not serrate
Apex of penial truncus	Wider than ventral plate basal width	Narrower than ventral plate basal width	Narrower than ventral plate basal width

**Table 2.** *Iandumoema smeagol* sp. n., measurements (in mm) of appendages of male paratype (MZUSP 67947) and female paratype (LES/UFSCar 0006299; in parentheses).

	Tr	Fe	Pt	Ti	Mt	Ta	Total
Leg I	0.3 (0.4)	3.9 (3.0)	1.1 (0.8)	2.9 (2.1)	4.8 (3.5)	2.5 (2.1)	15.5 (11.9)
Leg II	0.5 (0.3)	8.1 (5.3)	1.5 (1.3)	6.8 (5.1)	9.2 (6.1)	6.9 (5.2)	25.7 (23.3)
Leg III	0.6 (0.3)	5.4 (3.9)	1.3 (0.8)	3.1 (2.2)	5.5 (4.1)	2.3 (1.5)	18.2 (12.8)
Leg IV	1.1 (0.5)	6.9 (5.5)	2.0 (1.5)	5.2 (3.8)	7.4 (6.1)	2.2 (2.3)	24.8 (19.7)
Pedipalp	0.6 (0.4)	2.1 (1.5)	0.9 (0.8)	1.6 (1.1)	---	1.1 (0.9)	6.3 (4.7)

##### Description.

**Figure 2.**
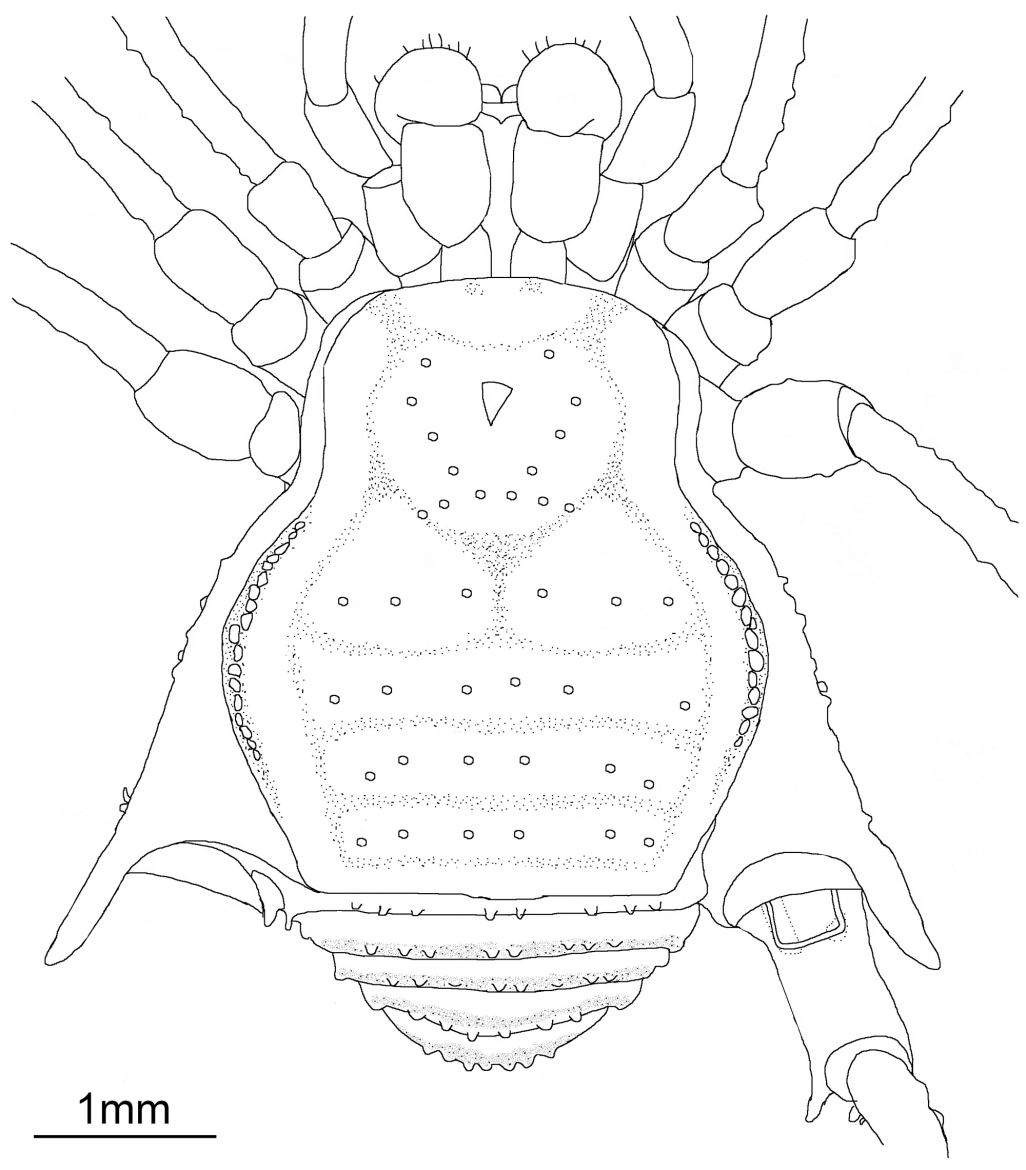
Drawing of *Iandumoema smeagol* sp. n. Male (holotype): habitus, dorsal view showing tubercles.

**Figure 3.**
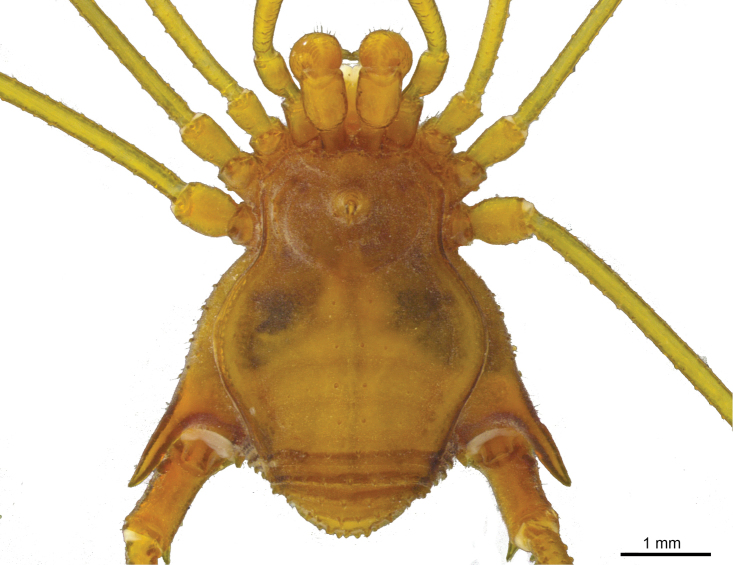
Photography of *Iandumoema smeagol* sp. n. Male (holotype): habitus, dorsal view.

**Figure 4.**
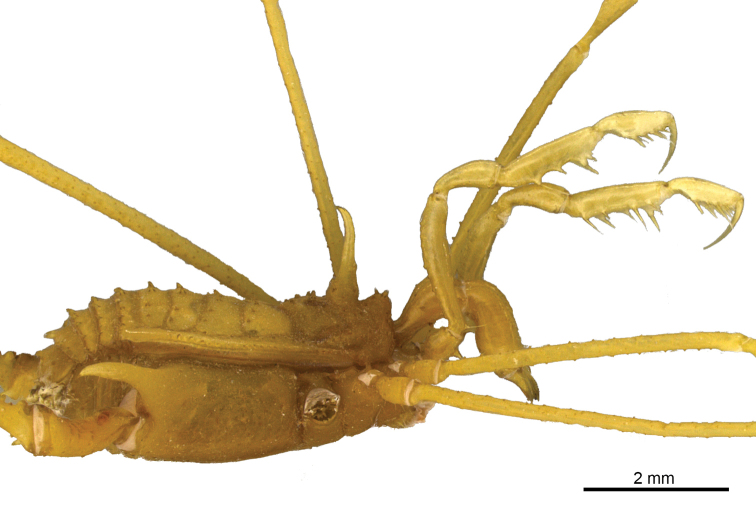
*Iandumoema smeagol* sp. n. Male (holotype): habitus, right lateral view.

Male: Dorsum (Figures 2, 3, 4): Measurements (paratype MZSP-67947): Dorsal scutum length 3.6; prosoma length 1.7; prosoma width 2.1; opisthosoma maximum width 3.1. Measurements of legs provided in Table 2. Frontal hump with five tubercles (paramedian pair largest), anterior margin of dorsal scutum with 4–5 tubercles on each side. Ocularium without eyes; with high upwardly directed spine, apex curved backwards. Each side of ocularium with 2-3 tubercles. Prosoma with 10 tubercles posterior to ocularium. Scutal area I divided, with three tubercles on each side; scutal area II with one transversal row of 6-7 tubercles; scutal areas III–IV each with seven tubercles, paramedian pair largest and pointed on all areas. Lateral margin of dorsal scutum with an external row of 21–24 tubercles from sulci I–IV and an internal one with 14–16 tubercles from sulci I–II. Posterior margin of dorsal scutum with 14 tubercles. Free tergite I with 11 tubercles; II with 12; III with 10 (three median larger). Anal operculum with an anterior row of seven tubercles and posterior part irregularly tuberculate.

**Figure 5.**
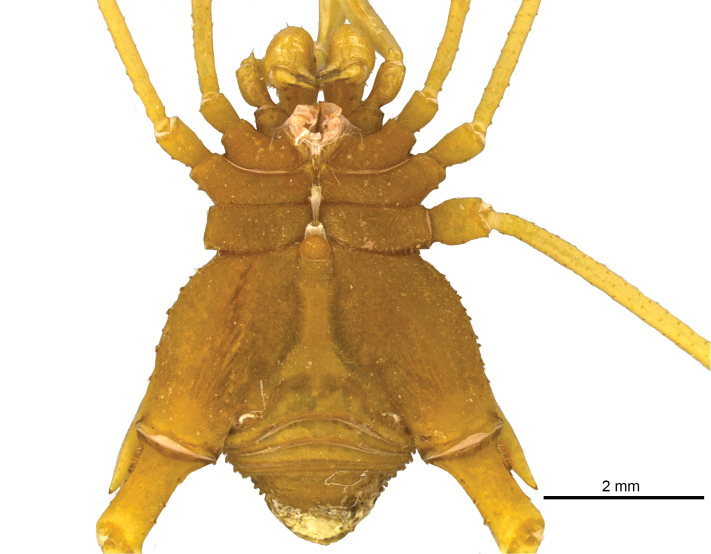
*Iandumoema smeagol* sp. n. Male (holotype): habitus, ventral view.

Venter (Figure 5): Coxa I with one median row of five anterior tubercles and four posterior tubercles; coxa II with 11 tubercles; coxa III with seven tubercles; coxa IV and stigmatic area irregularly tuberculate. Posterior margin of stigmatic area, free sternites, and anal opercle each with one row of tubercles.

Chelicera: Segment I elongated, bulla poorly defined, with four tubercles. Fixed finger with four equally sized teeth on the edge; movable finger with five teeth.

**Figure 6.**
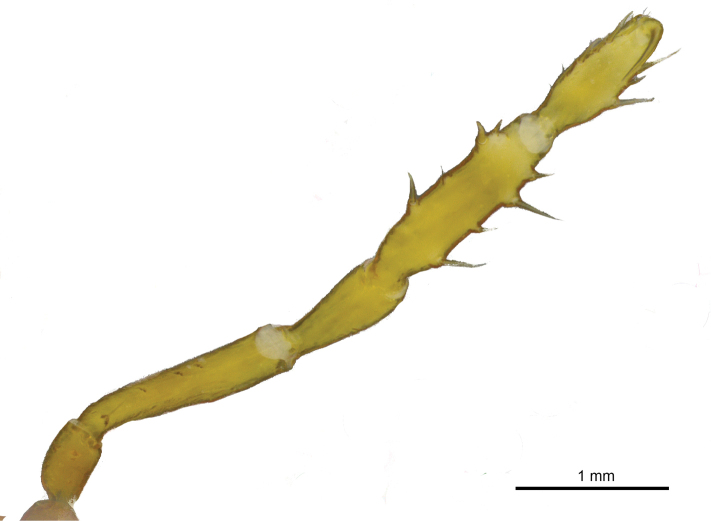
*Iandumoema smeagol* sp. n. Male (holotype): right pedipalp, ventral view.

Pedipalps (Figure 6): Slightly elongated. Coxa smooth. Trochanter with two dorsal and two ventral (ventro-mesal largest) tubercles. Femur with one ventro-basal large followed by three small tubercles. Patella smooth; tibial and tarsal spination: ectal and mesal IiIi.

**Figure 7.**
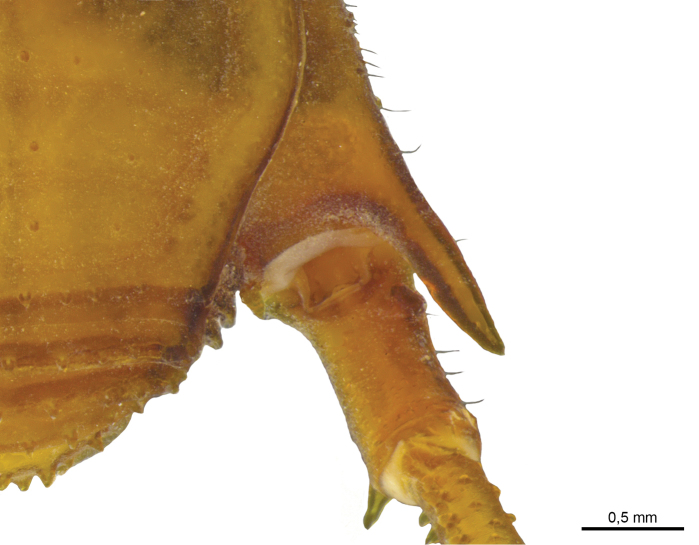
*Iandumoema smeagol* sp. n. Male (holotype): right trochanter IV, dorsal view.

**Figure 8.**
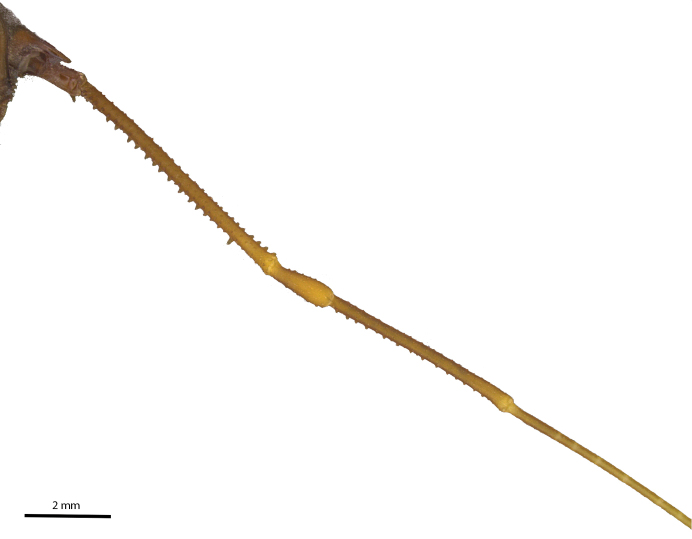
*Iandumoema smeagol* sp. n. Male (holotype): right leg IV, dorsal view.

**Figure 9.**
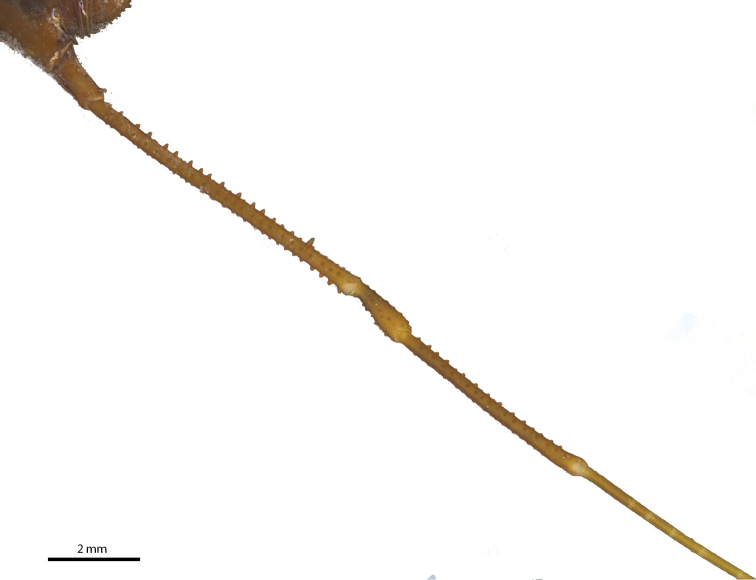
*Iandumoema smeagol* sp. n. Male (holotype): right leg IV, ventral view.

Legs (Figures 7, 8, 9, Table 2): Coxa I with two stout tubercles; II with one stout anterior tubercle, one median small and one stout posterior; III with two stout tubercles, one anterior fused with the posterior tubercle of coxa II and one posterior IV with scattered tubercles and with dorso-apical, slightly sigmoid, backwards-directed apophysis, with one retrolateral apical long apophysis (4× longer than wide). Trochanter I with two dorsal, one retrolateral and three ventral tubercles; II with four dorsal, two prolateral, one retrolateral and three ventral tubercles; III smooth dorsally, with three retrolateral and six ventral tubercles; IV dorsally smooth, with large basal prolateral submedian apophysis bearing one tubercle, and with four retrolateral (apical largest), and 12 small ventral tubercles. Femur–tibia III with small tubercles.

Femur IV straight, with two rows of irregular dorsal tubercles, two ventral rows of higher than others of same segment (twice as long as wide) tubercles on apex, one retrolateral row of irregular-sized tubercles, larger than other of same segment (third apical one largest), two enlarged dorso-apical tubercles (one prodorsal and one median). Patella IV with two ventral rows of tubercles, tuberculate on the sides, dorsally unarmed. Tibia IV with two rows of ventral tubercles of similar sizes. Basitarsus I of similar size as distitarsus. Tarsal segmentation: 6(3), 11(3), 6, 6.

**Figure 10.**
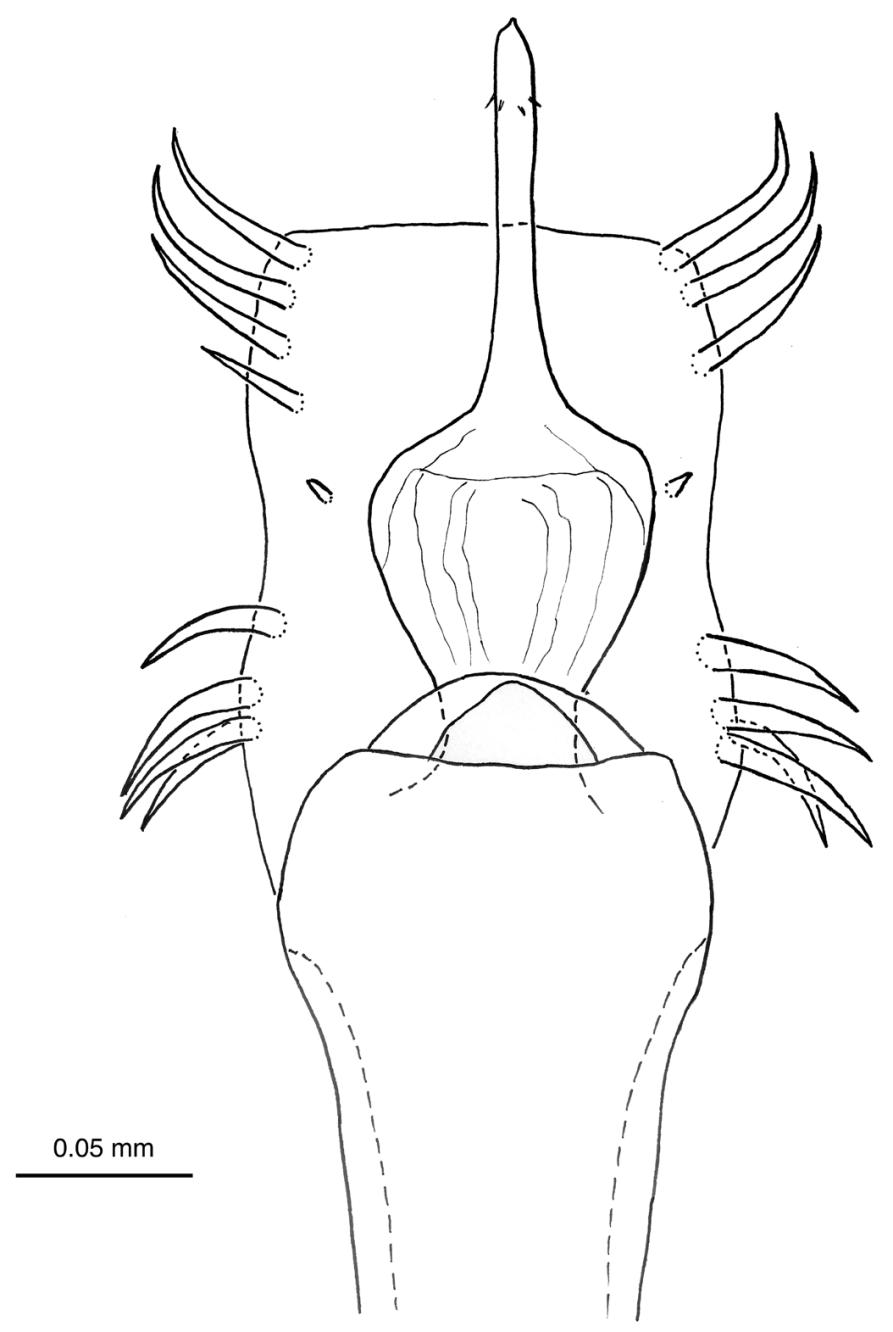
*Iandumoema smeagol* sp. n. Male (paratype, MZUSP 67947): distal part of penis, dorsal view.

**Figure 11.**
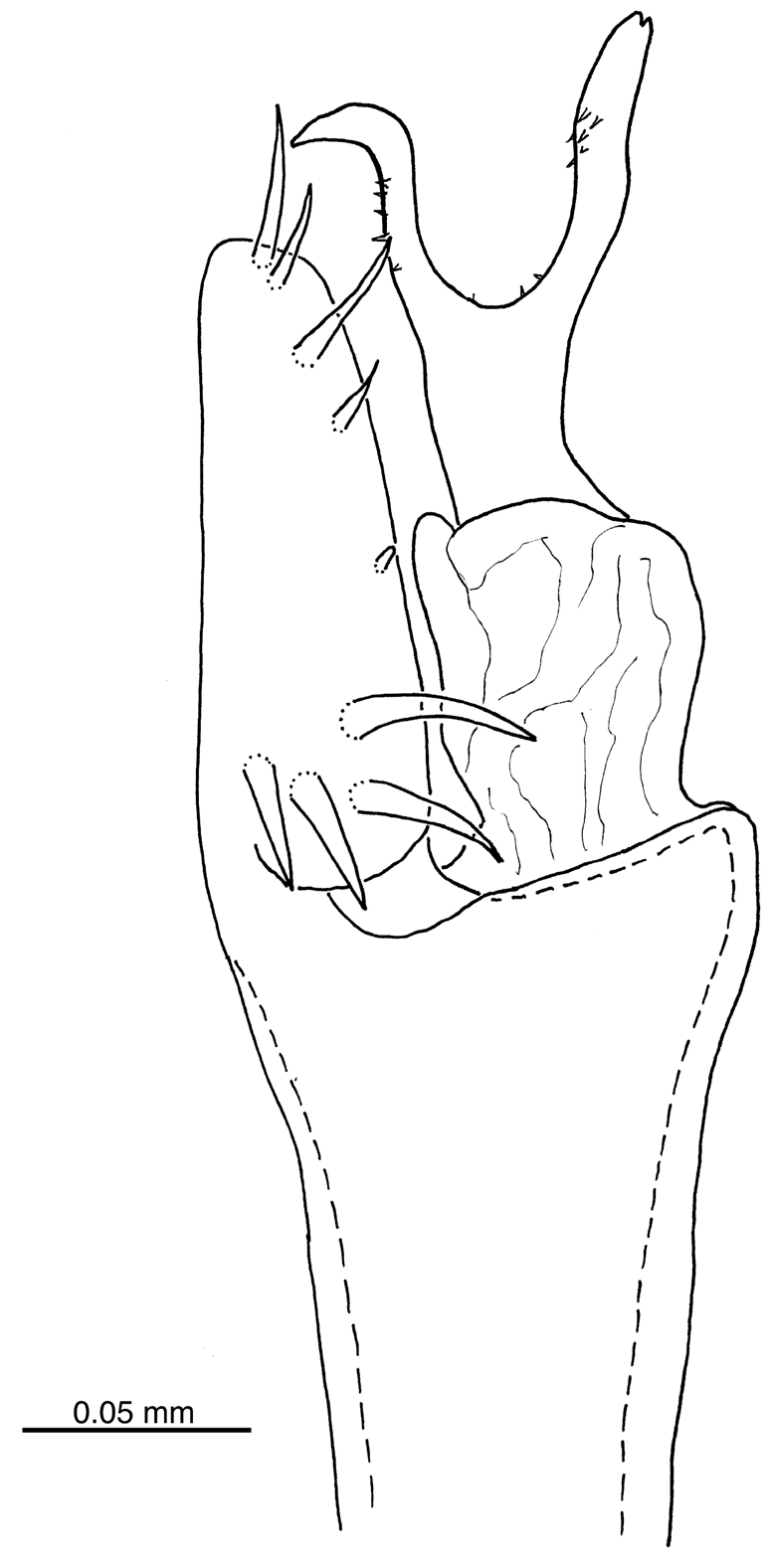
*Iandumoema smeagol* sp. n. Male (paratype, MZUSP 67947): distal part of penis, left lateral view.

Penis (paratype MZSP-67947, Figures 10, 11): Ventral plate subrectangular, with distal margin straight and a slight median constriction on the sides. Macrosetae: distal group with 3-4 on each side (C1–C4), basal one (C4, absent in the right) half-length of other three distal setae (similar sized, curved apically); median pair of setae (D1) placed more internally than groups A–C; basal group in arch (lateral view), formed by A1–3 and B1 (ventralmost), similar in length. Glans sac enlarged in the middle, stylus long and thicker than ventral process shaft; ventral process of glans without serrate distal margin, slender than and as long as stylus; both stylus and ventral process with ventromedian small microsetae.

##### Coloration

**Figure 12.**
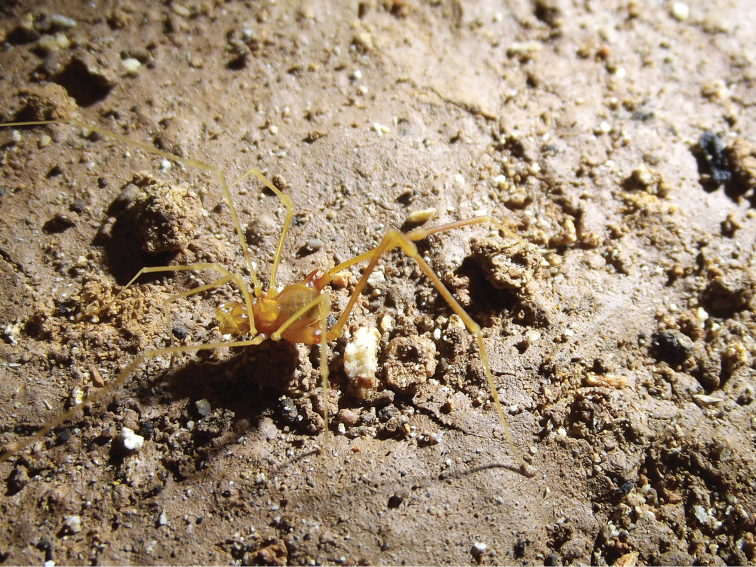
*Iandumoema smeagol* sp. n. Live male specimen foraging in its natural habitat, showing detail of the pale yellowish coloration.

(Figures 3, 12). Ethanol: Pale yellowish carapace with tip of tarsus and dorsal tibia whitish (Figure 3). Live specimens show a carapace with lighter coloration compared to the same part in the preserved specimen (Figure 12).

**Figure 13.**
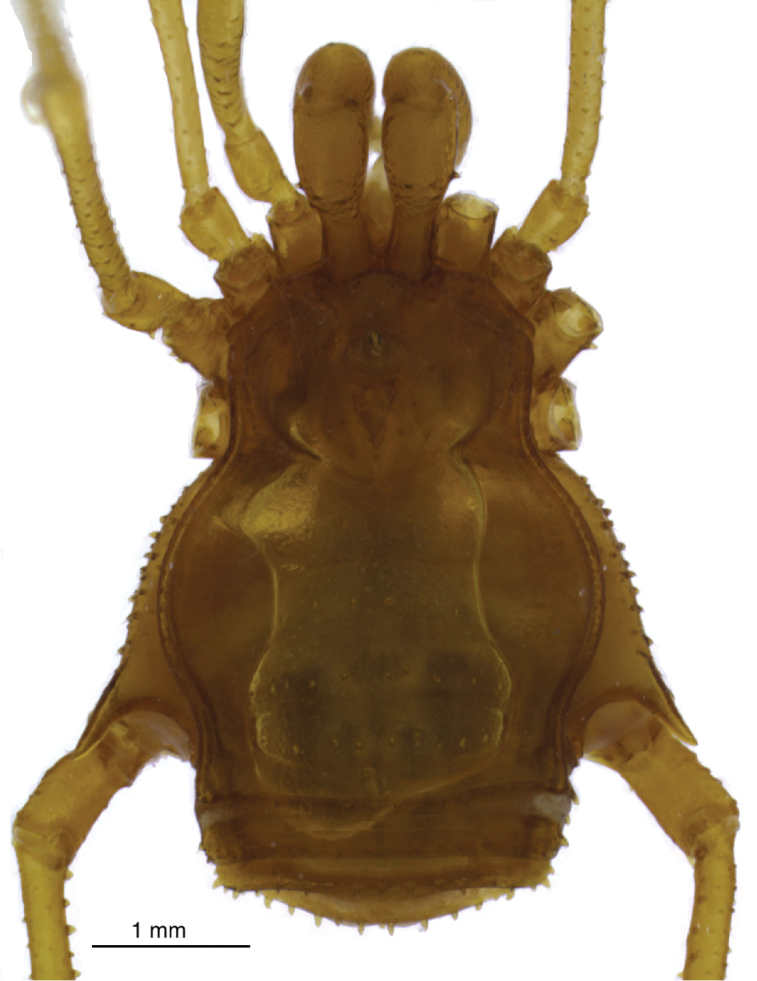
*Iandumoema smeagol* sp. n. Female (paratype, LES/UFSCar 6299): habitus, dorsal view.

Female (paratype, LES/UFSCar 0006299, Figure 13): Measurements: Dorsal scutum length 3.1; prosoma length 1.2; prosoma width 1.8; opisthosoma maximum width 2.4. Measurements of appendages are presented in Table 2. Only characteristics different from those of males are mentioned. Anterior margin of dorsal scutum with six tubercles on each side. Scutal area I with 3–5 tubercles on each side; scutal area II with eight; scutal area III with seven; scutal area IV with seven tubercles. Posterior margin of dorsal scutum with 13 tubercles. Free tergite I with 17; II with 17; III with 11 tubercles. Coxa IV with a shorter prolateral apophysis (half as long) than in male; trochanter IV with basal and median apophyses half as long or less than in male; tubercles on legs smaller than in male; femur IV with two enlarged dorso-apical tubercles.

##### Relationships.

*Iandumoema smeagol* sp. n. seems to be close related to *Iandumoema setimapocu* based on number of macrosetae on penis, four pairs on group A+B (six in *Iandumoema uai*) and apex of truncus narrower than ventral plate basal width. The shape of male apophysis on coxa IV is similar in both species, being obliquely directed, as also the presence of a submedian prolateral apophysis on male trochanter IV. However, a cladistics analysis is necessary to reveal well-supported relationships among *Iandumoema* species.

##### Distribution and natural history.

The occurrence of *Iandumoema smeagol* sp. n. in the limestone caves of Bambuí Group, more specifically in the boundaries of Serra do Espinhaço Plateau (Figure 1) shows that this region must be the eastern boundary distribution of the genus, the quartzite and the high altitudes of Serra do Espinhaço being the possible barriers. The results show that the genus *Iandumoema* only occurs in the northern Minas Gerais state, occupying an area of *ca.* 8,000 km^2^, and is restricted to hypogean environments, being exclusive to caves. This distribution range corroborates those presented by Hara and Pinto-da-Rocha (2008). Most specimens were collected in the aphotic zone of Toca do Geraldo cave; and only one individual was recorded in the Lapa do Santo Antônio cave. The minimum distribution range for *Iandumoema smeagol* sp. n. (or occurrence area) is of 4.6 km^2^. The specimen collected in the Lapa do Santo Antônio cave was on the rocky substrate, at the twilight zone and close to the entrance (less than 50 m away). In four visits at Toca do Geraldo, the opilionids were observed on the walls (rocky substrate) and few on the silt substrate, always close to water bodies (drainage or pools). Despite the observed guano piles (of hematophagous bats), not one individual was observed close to them. The adults show solitary habits; on one occasion, one individual was feeding in litter, apparently scavenging carcasses of invertebrates (Figure 12). In two occasions, active juveniles were observed on the walls while the adults showed a behavior comparatively more sedentary. In the four occasions, a total of 14 individuals were observed including adults and juveniles, always close to the cave stream, showing a low abundance. Apparently, the cave does not have dry galleries and/or conduits, showing high relative humidity of the air (*ca.* 80%) and temperature amplitude between 22 and 24 °C.

##### Troglomorphisms and conservation remarks.

As a result of their faunistic singularities and high endemism, hypogean environments are considered fragile. Besides their unique faunistic composition, the singularity of cave habitats is related to the presence of relicts, many times represented by troglobitic species. Gallão and Bichuette (2015) observed this tendency in a small area (24 km^2^) located at Chapada Diamantina, northeastern Brazil (at least 23 troglobitic species, most of them relict ones). Troglobitic species have unique sets of autapomorphies, such as eyes and melanistic pigmentation reductions allied to other troglomorphisms, such as pedipalps elongation in opilionids and other arachnids. A possible endemism in a karst area, which is threatened, was observed for *Iandumoema smeagol* sp. n. in addition to the accentuated autapomorphies. Projects for the installation of small hydroelectric dams and limestone extraction for cement production represent potential impacts on the immediate environment (M. E. Bichuette and R. Fonseca-Ferreira, pers. obs.). Moreover, the extent of occurrence area of the species (4.6 km^2^) allied to the deforestation in the cave surroundings must place this species in a threatened category considering the IUCN criteria (Vulnerable, VU or Endangered, EN). Long-term studies focusing population biology and distribution of *Iandumoema smeagol* sp. n. are urgent and fundamental to establish an effective conservation policy, including the creation of protected area(s).

## Supplementary Material

XML Treatment for Iandumoema smeagol
